# Early COVID-19 and protection from Omicron in a highly vaccinated population in Ontario, Canada: a matched prospective cohort study

**DOI:** 10.1186/s12879-024-10331-1

**Published:** 2025-02-08

**Authors:** Altynay Shigayeva, Christopher Kandel, Lubna Farooqi, Zoe Zhong, Anne-Claude Gingras, Brenda L. Coleman, Lois Gilbert, Wayne L. Gold, Maria Major, Tony Mazzulli, Samira Mubareka, Jelena Vojicic, Jingyan Yang, Pingping Zhang, Catherine Martin, Moe H. Kyaw, John M. McLaughlin, Allison McGeer

**Affiliations:** 1 Department of Microbiology, Sinai Health, Toronto, Canada; 2 Michael Garron Hospital, Toronto East Health Network, Toronto, Canada; 3 Department of Medicine, University of Toronto, Toronto, Canada; 4 Lunenfeld-Tanenbaum Research Institute, Sinai Health, Toronto, Canada; 5 Department of Molecular Genetics, University of Toronto, Toronto, Canada; 6 Dalla Lana School of Public Health, University of Toronto, Toronto, Canada; 7 University Health Network, Toronto, Canada; 8 Pfizer Canada, Kirkland, Canada; 9 Department of Laboratory Medicine and Pathobiology, University of Toronto, Toronto, Canada; 10 Sunnybrook Health Sciences Centre, Toronto, Canada; 11 Pfizer Inc, New York, USA

**Keywords:** SARS-CoV-2 infection, COVID-19, Vaccination, Re-infection

## Abstract

**Objectives:**

Predictions regarding the on-going burden of SARS-CoV-2, and vaccine recommendations, require an understanding of infection-associated immune protection. We assessed whether early COVID-19 provided protection against Omicron infection.

**Methods:**

We enrolled a cohort of adults in Ontario, Canada, with COVID-19 prior to October 2020 (early infection, EI), and a matched cohort with COVID-19 testing and a negative PCR (non-EI). Participants completed baseline surveys then surveys every two weeks until January 2023. Multivariable Cox regression was used to assess factors associated with COVID-19 infection during the first 14 months of Omicron.

**Results:**

Overall, 624 EI (70%) and 175 (77%) non-EI participants met criteria for analysis; 590 (95%) EI and 164 (94%) non-EI had received at least 2 COVID-19 vaccine doses prior to Omicron. Of 624 EI, 175 (28%) had one SARS-CoV-2 re-infection and 8 (1.3%) had two, compared to 84 (48%) non-EI participants with one, 5 (2.9%) with two and 1 (0.6%) with 3 infections (*P* < 0.0001). In multivariable analysis of risk factors for Omicron infection, the overall hazard ratio (HR, 95%CI) associated with EI was 0.56 (0.43–0.74); HRs for BA.1/2, BA.4/5 and mixed BA.5/BQ.1/XBB periods were 0.66 (0.45–0.97), 0.44 (0.28–0.68) and 0.71 (0.32–1.56). EI and BA.1/2 infection combined reduced later Omicron infection (HR 0.07 (0.03–0.21) compared to no prior infection. Older age, non-White ethnicity, no children in household, and lower neighbourhood income were associated with reduced risk of infection.

**Conclusions:**

In our highly vaccinated population, early SARS-CoV-2 infection was associated with a 44% reduction in symptomatic COVID-19 during the first 14 months of Omicron, providing significant protection against re-infection for more than 2 years.

**Supplementary Information:**

The online version contains supplementary material available at 10.1186/s12879-024-10331-1.

## Introduction

As of 12 December 2022, more than 97% of the world’s population had some protection from SARS-CoV-2 infection from vaccination and/or infection [1]. This has dramatically reduced the severity of COVID-19: the mortality rate of SARS-CoV-2 infection in June 2023 dropped to its lowest level since March of 2020 despite global relaxation of measures to prevent transmission [2]. Nonetheless, the estimated burden of severe COVID-19 continued to exceed that of influenza during 2023 and early 2024 [3, 4]. 

The future trajectory of the burden associated with SARS-CoV-2 infection depends on the magnitude and duration of protection afforded by past infection and vaccination [5, 6]. At least three meta-analyses have assessed the degree of protection against re-infection afforded by past infection [7–9]. Pre-Omicron, such protection remained high (> 75%) for at least 40 weeks. However, protection against Omicron was lower and declined over time, reaching ~ 40% at one year after infection. Data regarding the protection against BA.4/5 afforded by infection with ancestral viruses are sparse and conflicting [7, 10–12]. 

Key aspects of protection conferred by past SARS-CoV-2 infection are the extent to which natural immunity wanes over time, and the extent to which protection is reduced against new variants. Thus, an unavoidable limitation of published research remains the duration of follow-up; in addition, the switch in many countries from laboratory-performed polymerase chain reaction (PCR) to self-administered rapid antigen tests (RATs) limits the ability of population-based studies to reliably identify infections after March 2022 [12]. We followed matched cohorts of adults who were and were not infected with SARS-CoV-2 before 1 October 2020, until 31 January 2023, in order to estimate the protection against Omicron variants conferred by early infection with wild-type SARS-CoV-2.

## Methods

### Setting, design and participants

**Fig. 1:**
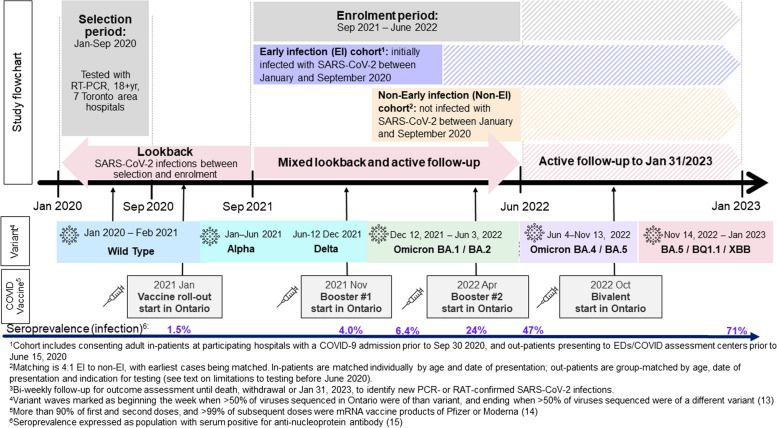
Flow chart of study procedures, variant evolution and vaccination in Toronto from January 2020 to January 2023

This prospective cohort study recruited in- and out-patients tested for SARS-CoV-2 at seven hospitals in the greater Toronto area, Ontario, Canada. Figure 1 displays the timing of cohort recruitment and follow-up in comparison to SARS-CoV2 variant evolution, vaccine programs and population infection seroprevalence in our population [13–15]. 

Variant waves are defined based on Ontario’s routine whole genome sequencing of Ontario specimens yielding SARS-CoV-2, as beginning the first week when more than 50% of sequenced viruses were of that variant, and ending the last week when more than 50% of sequenced viruses were of another variant/sub-variant [13]. Seroprevalence represents the population prevalence of anti-nucleoprotein antibody at labelled points in time [15]. PCR testing was limited to international travellers, contacts, healthcare workers and patients requiring hospitalization from March to early June 2020, then available free to all in assessment centres until January 2022; RATs were available beginning in the fall of 2021 and provided free to all at pharmacies from 9 February 2022 until late 2023.

Patients were enrolled into two cohorts: the early infection (EI) cohort, comprised of those infected with SARS-CoV-2 (i.e. positive PCR test from a nasopharyngeal swab) between 15 January and 15 June 2020, and the non-early infection (non-EI cohort), comprised of patients who presented with compatible symptoms but whose PCR tests were negative for SARS-CoV-2 (i.e. did not have an early infection), matched to the EI cohort on hospitalization status, age group, date of SARS-CoV-2 testing, and indication for testing (healthcare worker, contact of known case, or traveller). To meet sample size requirements, the infection dates of hospitalized cases and their matched controls (but not non-hospitalized cases and controls) were extended to September 30, 2020. The study was approved by Research Ethics Boards at all participating hospitals.

### Study procedures

Consenting participants were referred to a website to complete a baseline questionnaire including demographics, health conditions, medication use, adherence to public health recommendations, work status, and history of respiratory infection and COVID-19 testing from March 2020 to enrolment. Participants were then followed every two weeks until death, withdrawal, or 31 January 2023 to identify respiratory illnesses, PCR or RAT results, and to document receipt of COVID-19 vaccine doses. Participants reported all COVID-19 testing results, and completed illness reports for any acute respiratory or febrile illness. An added survey administered in the fall of 2022 asked for work status during the period January to May 2022. Per participant preference, questionnaires could be self-completed using the web-based application (with email reminders every two weeks), or administered by trained research personnel by telephone. Study questionnaires are found in the Supplementary material. The study also mailed RAT kits and kits to collect and submit saliva samples to the study for PCR testing to all participants at enrolment to ensure that testing was accessible.

### Primary outcome

The primary outcome was SARS-CoV-2 infection between 12 December 2021 and 31 January 2023, defined as a positive RAT performed by a participant or a health care provider, or a positive PCR test from a nasopharyngeal swab or saliva specimen submitted to the study laboratory, or reported by a participant from a specimen tested by a laboratory licensed by the province of Ontario. Reinfection was defined as a SARS-CoV-2 positive test with a specimen obtained ≥ 60 days after a previous positive specimen [16]. 

## Definitions

Immunocompromising conditions and medications were defined as previously described [17]. Statistics Canada postal code conversion files were used to identify participants’ neighbourhood household income quintiles based on the 2021 Census [18]. Work status was categorized as essential if participants reported working full- or part-time in-person outside of the home. Other work status comprised those working only at home or who were students, retired or unemployed. We asked participants to self-report their daily frequency of hand-to-face habits (rubbing eyes, biting nails or cuticles, or habitually putting fingers into the mouth or nose) [19, 20]. 

### Statistical analysis

Participant characteristics were summarized for EI and non-EI groups for those with complete baseline, every 2 week and vaccination questionnaires. Categorical data were compared using Chi-square or Fisher’s exact tests. Continuous variables were compared using the Mann-Whitney U test.

Unadjusted SARS-CoV-2 incidence density during each variant wave was summarized as the number of infections per 1000 person-days from 1 October 2020 to 31 January 2023. Differences in incidence rates in EI vs. non-EI participants were reported as incidence rate ratios (IRR) with 95% confidence intervals [21]. 

Univariate Cox regression was used to estimate the hazard ratio of infection comparing EI and non-EI groups during the Omicron wave. Multivariable Cox regression models included adjustment for vaccine doses (as a time-varying variable), age (with restricted cubic spline), severity of 2020 illness, ethnicity, education, neighbourhood income quintile, and presence of children in the household. Secondary analyses used piecewise exponential models with vaccine doses (as a time-varying variable), a spline on time since last dose to account for non-linear decay of protection, an interaction between vaccine dose number and time from last vaccine dose, and time from vaccination set to 1000 days for unvaccinated persons [22, 23]. 

Sensitivity analyses considered only symptomatic Omicron infections as the outcome, excluded participants with eligible infection episodes after May 2020, and considered Omicron infections diagnosed by PCR only. We also assessed the extent to which EI affected infection after the BA.1/2 wave in participants with and without Omicron infection during the BA.1/2 wave.

### Sample size

Our sample size calculation estimated that with a two-sided alpha of 0.05, a power of 80%, a 4:1 ratio of EI to non-EI participants and 700 total participants, we could detect as statistically significant a 36% reduction in infection (from 30 to 19%) in EI versus non-EI participants. We attempted to enrol 800 participants with EI, with equal numbers of hospitalized and non-hospitalized patients, and 200 controls.

## Results

### Study cohort

**Fig. 2:**
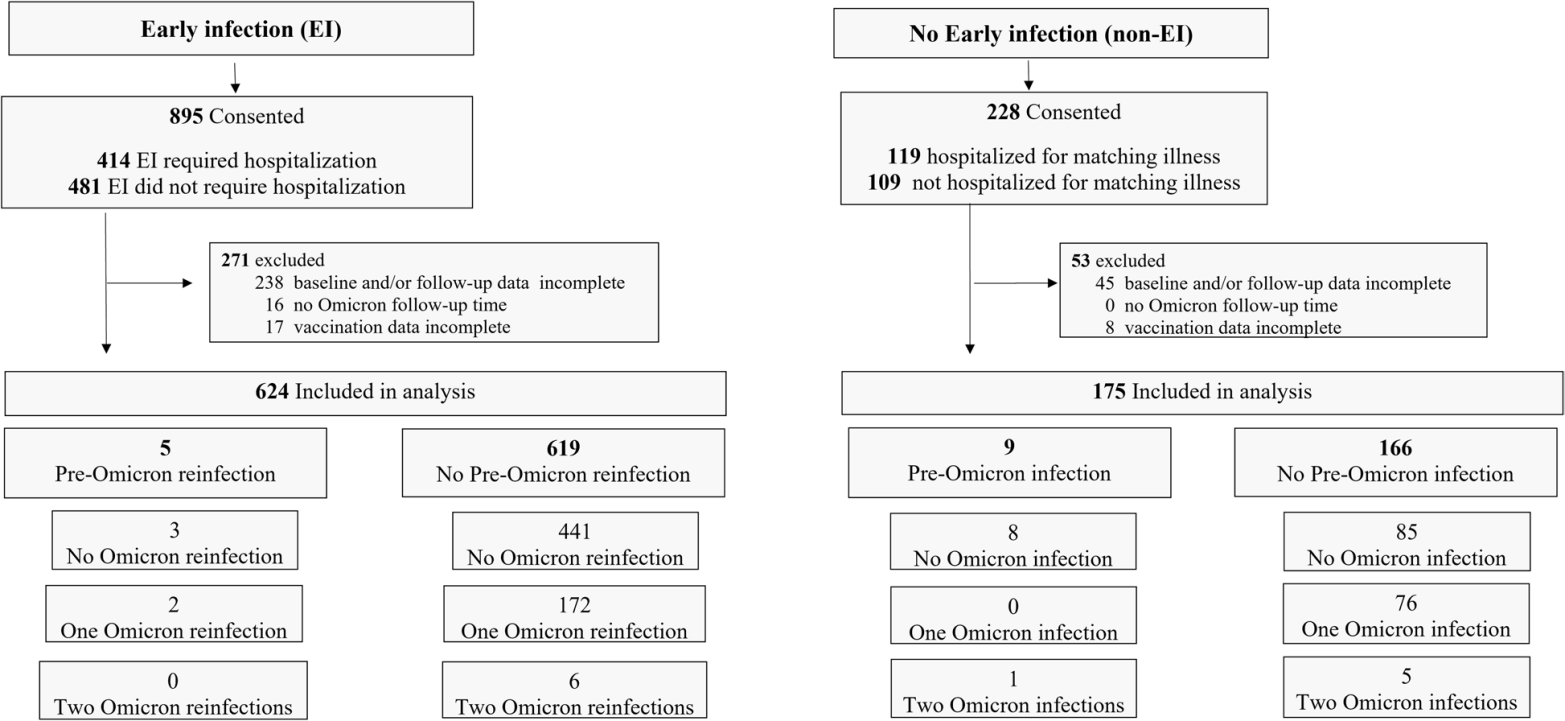
Flow chart of study consent, enrollment and follow-up. Note that 2 participants enrolled as non-EI were reclassified as EI because they reported in their baseline questionnaire having had a SARS-CoV-2 infection after their test-negative episode defining eligibility but before 30 September 2020

Overall, 1123 patients were enrolled (893 as EI, 230 as non-EI). Two persons enrolled as controls reported a SARS-CoV-2 infection before 30 September 2020 but after their test-negative episode of disease; these participants were re-coded as EI participants. Of all enrolled persons, 624 EI (70%) and 175 (77%) non-EI participants had complete data (Fig. 2).

Compared to non-EI participants, EI participants were more likely to live in low-income neighbourhoods, less likely to be Caucasian and to have a university education, and more likely to be essential workers (Table 1). The excess of underlying illness in non-EI participants was seen only in the outpatient group and included persons with underlying asthma, other respiratory, and cardiac disease (data not shown).

**Table 1:** Characteristics of the study cohorts with and without RT-PCR confirmed SARS-CoV-2 infection prior to September 30, 2020 (early infection)

	With Early Infection^1^ (*N*=624)	No Early Infection^1^ (*N*=175)	*P* value
**Sex at birth, female (N, %)**	327 (52.4%)	96 (54.9%)	0.63
**Age in years (median, IQR)**	55.7y (42.6-65.1)	56.4y (39-68.2)	0.80
**Age group (N, %)**			
18-49 years	233 (37.3%)	69 (39.4%)	0.06
50-64 years	224 (35.9%)	47 (26.9%)	
≥65 years	167 (26.8%)	59 (33.7%)	
**Underlying illness**^**2**^** (N,%)**			
Any	216 (34.6%)	76 (43.4%)	0.03
Diabetes mellitus	108 (17.3%)	25 (14.3%)	0.27
Cardiac disease	64 (10.3%)	28 (16%)	0.04
Immunocompromised	38 (6.1%)	11 (6.3%)	1.0
**Severity of eligible illness in 2020 (N, % hospitalized)**	279 (44.7%)	91 (52.0%)	0.09
**Children in household (N, % with children ≤17 yr)**	169 (27.1%)	58 (33.1%)	0.12
**Ethnic Background (N, %)**			
Caucasian (White)	236 (37.8%)	93 (53.1%)	0.001
Asian (East, Southeast and South)	187 (30.0%)	42 (24.0%)	
Black	79 (12.7%)	10 (5.7%)	
Other	122 (19.6%)	30 (17.1%)	
**Education (N, %)**			0.001
Secondary school or less	154 (24.7%)	36 (20.6%)	
College level diploma or certificate	207 (33.2%)	38 (21.7%)	
Undergraduate university degree	169 (27.1%)	60 (34.3%)	
Graduate or professional degree	85 (13.6%)	39 (22.3%)	
**Neighbourhood income quintile (N, %)**			0.005
1-lowest	198 (31.7%)	43 (24.6%)	
2	150 (24.0%)	33 (18.9%)	
3	110 (17.6%)	30 (17.1%)	
4	74 (11.9%)	23 (13.1%)	
5-highest	92 (14.7%)	46 (26.3%)	
**Work status during Omicron wave**^**3**^ **(N,%)**			<.001
Essential worker	334 (53.5%)	64 (36.6%)	
Other	287 (46.0%)	111 (63.4%)	
**Hand-to-Face habits**^**4**^			0.04
None	175 (28.0%)	33 (18.9%)	
≤5 times per day	342 (54.8%)	106 (60.6%)	
≥6 times per day	101 (16.2%)	35 (20.0%)	
**COVID-19 vaccine doses received as of 11 Dec 2021**^**5**^			0.02
Not vaccinated	28 (4.5%)	9 (5.1%)	
One dose	6 (1.0%)	2 (1.1%)	
Two doses	466 (74.7%)	111 (63.4%)	
Three doses	124 (19.9%)	53 (30.3%)	

^1^Note that totals in columns may differ from overall total because of missing data for some variables. Data are complete for all variables except education (missing in 9 (1.4%) of EI and 2 (1.1%) of Non-EI participants), work status during Omicron (missing in 3 (0.5%) of EI and none of non-EI participants), and hands-to-face habits (missing in 6 (1.0%) of EI and 1 (0.6%) of no-EI participants)

^2^Any underlying illness predisposing to complications of viral respiratory illness [24]. Diabetes mellitus and underlying cardiac disease were the only two conditions present in at least 10% of participants. Immunocompromised includes both immunocompromising illness and therapy [10]

^3^Work status was categorized as essential if participants reported working full- or part-time in-person outside of the home. Others comprised those working only at home or who were students, retired or unemployed

^4^Hand-to-face habits were defined as rubbing eyes, biting nails or cuticles or habitually putting fingers into the mouth or nose

^5^December 12, 2021 represents the start of the Omicron wave in the population area (see text); 50% of participants without EI had received a 3 dose as of 17 December; while 50% of those with EI had received their 3 dose by 28 December

At the start of Omicron on 12 December 2021, 94.5% (590/624) EI participants and 93.7% (164/175) non-EI participants had received at least two doses of the vaccine. There was no difference in the timing of the first two doses of vaccine among EI and non-EI participants (median dates of second dose receipt were 21 June and 18 Jun 2021, respectively). Non-EI participants received their third doses slightly earlier than EI participants (median dates of receipt 18 December 2021 and 4 January 2022, respectively *P* < .0001) such that non-EI recipients were more likely have received a third dose on 12 December 2021 (30.3% versus 19.9%, *P* = 0.003; Table 1). Detailed timing of vaccine doses in the two cohorts is shown in Supplementary Figure 2. At the end of study follow-up, 73.4% (458/624) of EI participants and 76.7% (134/175) of non-EI participants had received a third dose of a COVID-19 vaccine (*P* = 0.26), 32% (200/624) of EI and 51% (89/175) of non-EI participants had received a fourth dose , and 9.9% (62/624) of EI and 22% (38/175) non-EI had received a fifth dose (*P* < .0005 for both).

### SARS-CoV-2 infections during follow-up

**Fig. 3:**
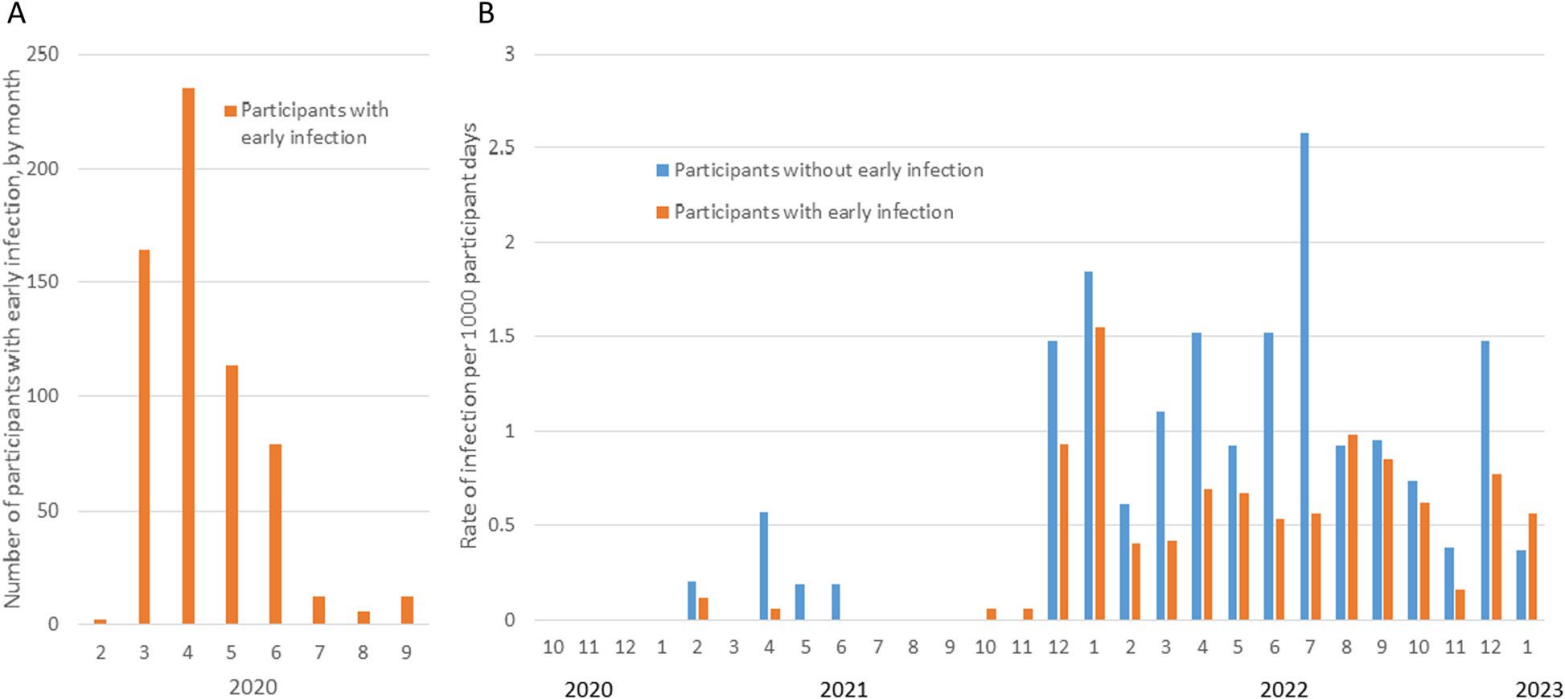
Incidence rate of PCR- or RAT-confirmed SARS-COV-2 infections comparing participants in the early infection (EI) cohort to those in the non-early infection (non-EI) cohort. Panel **A** displays the distribution of month of diagnosis in the early infection cohort in 2020, while Panel **B** displays the infection rate by cohort from October 2020 to January 2023

Participant infections and receipt of vaccine doses were tracked over a median of 31.8 (IQR 19.9–33.1) months. Among the 799 participants, 273 individuals had a total of 288 episodes of SARS-CoV-2 infection between 1 October 2020 and 30 January 2023 (Figs. 2 and 3). Of 624 EI participants, 175 (28%) had one SARS-CoV-2 re-infection episode and 8 (1.3%) had two re-infection episodes during follow-up. Among 175 non-EI participants, 84 (48%) had one infection, 5 (2.9%) had two infections and 1 (0.6%) had 3 infections (*P* < 0.0001 compared to EI participants).

Overall, 269/288 (93.4%) identified infections during study follow-up were symptomatic, and 7 (2.4%) required hospitalization. Symptomatic infections comprised 169/183 (92%) of first re-infections among EI participants, and 87/90 (97%) of first infections among non-EI participants (*P* = 0.19); hospitalizations occurred in 6/183 EI (3.3%) re-infections and 1/90 (1.1%) non-EI infections (*P* = 0.32). Overall, 14 infections occurred prior to the Omicron wave: these participants were excluded from the analysis of risk factors for infection during Omicron.

### Factors associated with SARS-CoV-2 infection during Omicron

**Fig. 4:**
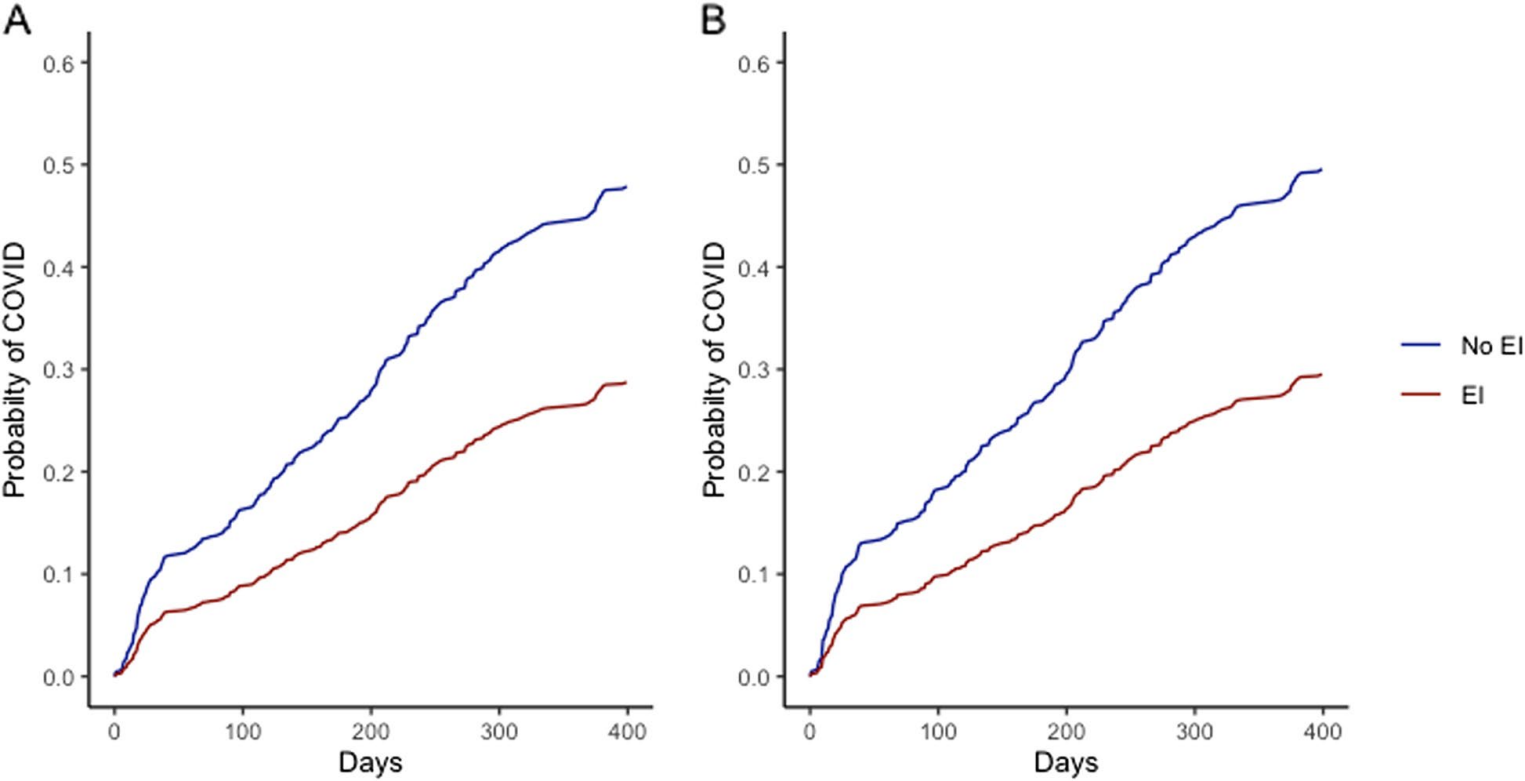
Cumulative probability of SARS-CoV-2 infection during Omicron, comparing participants in the early infection (EI) cohort to those in the non-early infection (non-EI) cohort. Panel **A** displays the unadjusted and Figure **B** the analysis adjusted for age, severity of 2020 illness, ethnicity, education, neighbourhood income quintile, presence of children in the household and COVID-19 vaccination

In univariate Cox regression, infection during the Omicron wave was less common in those with EI, in older adults, and in persons who had more vaccine doses during the Omicron period (Fig. 4A; Table 2). Infection was more common in persons originally treated as outpatients, in essential workers, in those living in higher income neighbourhoods, and in persons with frequent hand-to-face habits.

**Table 2:** Univariate and multivariable analysis of factors associated with SARS CoV-2 infection in study participants from 12 December 2021 to end of the study (31 January 2023)

	Rate per 1000 participant days	Univariate Cox Regression		Multivariable Cox Regression	
	Rate (95% CI)	Hazard ratio, 95% CI	*P* value	Hazard ratio 95% CI	*P* value
**Early SARS-CoV-2 infection**^**1**^					
No (non-EI)	1.75 (1.41-2.18)	Reference		Reference	
Yes (EI)	0.86 (0.74-0.99)	0.49 (0.38-0.64)	<.0001	0.56 (0.43-0.74)	<.001
**Sex at birth**					
Male	0.94 (0.78-1.13)	Reference			
Female	1.09 (0.93-1.29)	1.16 (0.90-1.48)	0.25	-	
**Age group**					
18-49 years	1.40 (1.17-1.67)	Reference			
50-64 years	0.91 (0.73-1.13)	0.66 (0.50-0.87)	0.0001	NE^6^	0.022
≥65 years	0.72 (0.55-0.93)	0.52 (0.38-0.71)			
**Underlying illness**					
Any^2^	0.94 (0.76-1.15)	0.87 (0.68-1.13)	0.31	-	
Diabetes mellitus	0.84 (0.61-1.15)	0.79 (0.56-1.12)	0.18		
Cardiac disease	0.80 (0.54-1.18)	0.76 (0.50-1.15)	0.20		
Immunocompromised	1.27 (0.79-2.04)	1.24 (0.76-2.03)	0.38		
**Severity of 2020 illness**					
Hospitalized	0.82 (0.68-1.00)	Reference		-	
Not hospitalized	1.21 (1.03-1.41)	1.46 (1.14-1.87)	0.003		
**Children (≤17yrs) in household**					
No	0.85 (0.73-1.00)	Reference		Reference	
Yes	1.53 (1.25-1.86)	1.77 (1.37-2.27)	<.0001	1.60 (1.19-2.15)	0.002
**Ethnic Background**					
Caucasian	1.29 (1.08-1.53)	Reference		Reference	
Asian (East/SE/South)	0.88 (0.69-1.12)	0.68 (0.51-0.92)	0.006	0.57 (0.41-0.79)	<.001
Black	0.63 (0.40-0.99)	0.50 (0.31-0.80)		0.57 (0.34-0.95)	0.03
Other	0.94 (0.70-1.25)	0.73 (0.52-1.03)		0.70 (0.49-1.00)	0.05
**Education**					
Secondary school or less	0.64 (0.47-0.87)	Reference		Reference	
College/diploma/certificate	0.94 (0.75-1.18)	1.46 (1.00-2.14)	0.0002	1.32 (0.09-1.95)	0.16
Undergraduate university degree	1.31 (1.07-1.61)	2.03 (1.40-2.94)		1.59 (1.08-2.32)	0.02
Graduate or professional degree	1.42 (1.08-1.85)	2.19 (1.46-3.29)		1.52 (0.99-2.32)	0.06
**Neighbourhood income quintile**					
1-lowest	0.71 (0.54-0.92)	Reference		Reference	
2	0.92 (0.71-1.20)	1.30 (0.89-1.88)	0.001	1.27 (0.88,1.83)	0.20
3	1.15 (0.87-1.51)	1.61 (1.10-2.36)		1.46 (0.99-2.14)	0.06
4	1.57 (1.17-2.11)	2.19 (1.47-3.25)		1.95 (1.31-2.91)	0.001
5 – highest	1.26 (0.97-1.65)	1.77 (1.22-2.58)		1.43 (0.96-2.12)	0.08
**Work status, Omicron**^**3**^					
Other	0.88 (0.73-1.06)	Reference		Reference	
Essential worker	1.17 (1.00-1.38)	1.33 (1.04-1.70)	0.02	1.29 (0.98-1.68)	0.07
**Hand-to-face habits**^**4**^					
None	0.74 (0.56-0.97)	Reference			
≤5 times per day	1.12 (0.96-1.31)	1.51 (1.10-2.06)	0.02	-	
≥6 times per day	1.25 (0.95-1.64)	1.68 (1.14-2.47)			
**COVID-19 vaccine doses before Omicron**^**5**^					
Not vaccinated	1.28 (0.73-2.25)	Reference		-	
One dose	0.53 (0.13-2.13)	0.42 (0.09-1.89)	0.46		
Two doses	0.99 (0.86-1.13)	0.78 (0.43-1.39)			
Three doses	1.18 (0.89-1.57)	0.93 (0.49-1.75)			
**Number of vaccine doses during Omicron**^**5**^					
None	1.67 (1.40-1.99)	Reference		NE^7^	
One dose	1.03 (0.85-1.24)	0.62 (0.48-0.81)	<.0001		
Two doses	0.38 (0.25-0.60)	0.23 (0.15-0.38)			
Three doses	0.21 (0.08-0.55)	0.13 (0.05-0.34)			
**Time since most recent vaccine dose **				NE^7^	
Not vaccinated	1.32 (0.73-2.38)	NA			
≥120 days	1.12 (0.97-1.30)	Reference			
75 - 119 days	0.97 (0.69-1.37)	0.95 (0.64-1.41)	0.78		
14-74 days	0.75 (0.56-1.00)	0.71 (0.50-1.01)	0.06		

*Abbreviations*: *SE* southeast, *FT* Full-time, *PT* part time, *CI* Confidence Interval, *NE* not estimable

^1^Early infection is defined as an RT-PCR confirmed SARS-CoV-2 infection between January 24 and 1 September 30, 2020

^2^Any underlying illness predisposing to complications of viral respiratory illness [24]. Diabetes mellitus and underlying cardiac disease were the only two conditions present in at least 10% of participants. Immunocompromised includes both immunocompromising illness and therapy [10]

^3^Work status was categorized as essential if participants reported working full- or part- time in-person outside of the home. Other comprised those working only at home or who were students, retired or unemployed

^4^Hand-to-face habits were defined as rubbing eyes, biting nails or cuticles or habitually putting fingers into the mouth or nose

^5^The start of the Omicron wave in the population area was December 12, 2021 (see text); vaccine dates are lagged by 14 days

^6^In multivariable analysis, age was modelled with a restricted cubic spline; the hazard ratio per decade of age was 0.82 (95% CI0.66–0.90)

^7^Vaccine doses were modelled as time varying in the multivariable analysis such that direct estimates are not available

In multivariable analysis, the hazard ratios associated with early COVID-19 infection did not change relative to univariate analysis (Fig. 4B; Table 2). In Cox regression analysis the hazard ratio for those with early infection versus those without was 0.56 (95%CI 0.43–0.74), in the piecewise exponential model, the incidence rate ratio was 0.57 (95%CI 0.44–0.75). In multivariable sensitivity analyses, the hazard ratio associated with prior infection was 0.52 (95%CI 0.40–0.69) when only symptomatic infections were included, 0.55 (95%CI 0.37–0.82) when only PCR-confirmed infections were included, and 0.54 (95%CI 0.39–0.74) when only EIs occurring before 31 May 2020 were included. Other factors associated with infection during the Omicron wave included younger age, being ethnically White, higher neighbourhood income, and living in a household with children (Table 2).

### Early infection and risk of infection with SARS-CoV-2 variants

Incidence rates of COVID-19 in EI and non-EI participants during periods of activity due to different variants, as well as the hazard ratio associated with infection in different subvariant periods in multivariable Cox regression analysis are shown in Table 3. Protection was highest against infections in the pre-Omicron era; estimates of reductions in IRR and adjusted infection hazard ratios during periods of activity with different predominant Omicron variants were not statistically significantly different. Early infection and infection during the BA.1/2 wave appeared to independently increase protection against later Omicron infections (Table 4): during the period from June 2022-January 2023, compared to those with no infection, the hazard ratio for a COVID-19 re-infection persons with early COVID-19 was 0.47 (95%CI 0.32–0.68), while that for persons with both an early infection and a BA.1/BA.2 infection was 0.07 (95%CI 0.03–0.21).

**Table 3:** Incidence of Omicron infection by time period^1^ in those with and without RT-PCR confirmed SARS-CoV-2 infection prior to 30 September 2020 (early infection)

	Early Infection (EI)	No Early Infection (non-EI)	EI vs. non -EI IRR (95% CI)	EI vs. Non-EI Hazard Ratio^2^ (95% CI)
**Prior to Omicron**				
Number of COVID-19 infections	5	9		
Participant-days	369,651	101273.7		
COVID-19 rate/1000 participant days	0.014	0.089	0.15 (0.05–0.45)	
**Omicron BA.1/BA.2**				
Number of COVID-19 infections	91	40		
Participant-days	107,331	30,142		
COVID-19 rate/1000 participant days	0.85	1.33	0.64 (0.44–0.93)	0.66 (0.45–0.97)
**Omicron BA.4/BA.5**				
Number of COVID-19 infections	68	38		
Participant-days	96,844	26,221		
COVID-19 rate/1000 participant days	0.70	1.45	0.48 (0.33–0.72)	0.44 (0.28–0.68)
**Mixed BA.5/BQ1.1/XBB**				
Number of COVID-19 infections	27	10		
Participant-days	44,859	11,817		
COVID-19 rate/1000 participant days	0.60	0.85	0.71 (0.34–1.47)	0.71 (0.32–1.56)

^1^Time periods are defined as follow: prior to Omicron, 25 January 2020–11 December 2021; Omicron BA1/BA.2, 12 December 2021–3 Jun 2022; Omicron BA4/BA5, 4 June 1–13 November 2022; and mixed BA.5/BQ1.1/XBB, 14 November 2022–31 January 2023 (end of study)

^2^Hazard ratios from the primary analysis: multivariable Cox regression adjusted for age, COVID-19 vaccination, severity of 2020 illness, neighborhood income quantile, ethnicity, education, work status during omicron and presence of children in the household

**Table 4:** Incidence of SARS-CoV-2 from 4 June 2022 to 31 January 2023 (due to BA.4/5 and later subvariants) in those with and without RT-PCR confirmed SARS-CoV-2 infection prior to 30 September 2020 (early infection)

	Early Infection (EI)	No Early Infection (non-EI)	EI vs. non -EI IRR (95% CI)	EI vs. Non-EI Hazard Ratio^2^ (95% CI)
**No BA.1/BA.2 infection**				
Number of COVID-19 infections	89	42		
Participant-days	121,623	28,779		
COVID-19 rate/1000 participant days	0.73	1.46	0.50 (0.35–0.72)	0.47 (0.32–0.68)
**With BA.1/BA.2 infection**^**1**^				
Number of COVID-19 infections	4	5		
Participant-days	20,665	9413		
COVID-19 rate/1000 participant days	0.19	0.53	0.36 (0.10–1.36)	0.20 (0.03–1.21)

^1^Comparing participants with no early or BA.1/BA.2 infection (42 cases during 28779 participant days) to those with both an early infection and a BA.1/BA.2 infection (4 cases during 20665 participant days), the IRR is 0.13 (95%CI 0.04–0.34) and the hazard ratio is 0.07 (95%CI 0.03–0.21)

^2^Hazard ratios from the primary analysis: multivariable Cox regression adjusted for age, COVID-19 vaccination, severity of 2020 illness, neighborhood income quantile, ethnicity, education, work status during omicron and presence of children in the household

## Discussion

In this cohort study in a highly vaccinated population, infection with SARS-CoV-2 during the first months of the pandemic was associated with an estimated reduction in subsequent infection risk of 80% during the pre-Omicron period, and 30–50% during the Omicron wave. The risk reduction appeared to be somewhat lower during periods of co-circulation of BA.5, BQ.1, XBB-related Omicron sub-lineages, but the confidence limits on this estimate do not permit conclusions about whether protection was declining over time/as new Omicron sub-lineages emerged.

Evidence regarding the additional degree of protection against symptomatic Omicron infection afforded by early ancestral infection in vaccinated individuals is mixed. Most studies suggest waning protection over time to 30–40% at 40–50 weeks [7], and at least two studies suggest little protection from pre-Omicron infection against BA.4/5 or XBB [11, 25]. However, Carazo et al. reported apparently stable protection of ancestral infection against BA.2 of approximately 40% over 58 weeks [6]. Some of the variability may be apparent only, as point estimates appear different but confidence limits overlap; [8, 11, 26] some may also be explained by differences in participant age, order of infection versus vaccination, or rates of undetected mild/asymptomatic infections in different populations [27, 28].

As others have identified, vaccine efficacy against any infection during Omicron was not dependent on the number of prior doses of vaccine received, but rather dependent on how recently a dose had been received [29–32]. Our study does not permit the assessment of protection against hospitalization or death, where the number of vaccine doses may be of greater importance [1].

The differences between the two cohorts in ethnicity, education, and neighbourhood income were to be expected, given the known higher rate of SARS-CoV-2 infection in economically marginalized communities in Toronto and elsewhere during the first months of the pandemic: essential workers were also at higher risk of COVID-19 during this period [33–35]. The excess of non-EI participants with underlying illness in persons with COVID-19 managed as outpatients may have occurred because persons with underlying cardio-respiratory conditions were more likely than others to have symptoms qualifying them for testing.

During the pandemic, the Canadian and Ontario governments introduced a number of policies attempting to mitigate the increased risk of COVID-19 associated with population inequities, including income support, eviction moratoria, and 3-day paid sick leave [36–38]. Although a population-level analysis suggested little effect of these policies on inequalities in hospitalization and deaths due to COVID-19 early during the Omicron wave [39] our data demonstrating that Omicron infections occurred more frequently among more highly educated participants of Caucasian descent living in higher-income neighbourhoods suggests that these may have had some impact. However, we cannot rule out the possibility that individuals in less vulnerable populations changed their behaviour and accepted an increased risk of infection before individuals in more vulnerable groups chose to. Also, it seems likely that adherence to risk-reducing behaviours (e.g. reducing contacts) explains the reduced risk of infection in older adults. Serology data from across Canada also demonstrates that the presence of infection-induced antibodies to SARS-CoV-2 is consistently lower among older versus younger adults [15, 40].

The availability of individual level data regarding potential risk factors, and the active follow-up with testing requested with any symptoms is a strength of this study. However, there are also limitations. Although we attempted to match our cohorts, and adjust for factors likely to affect testing practices or COVID-19 risk, we could not capture all potentially important factors. For instance, we attempted to measure self-reported adherence to public health measures, but found significant differences in responses at different time points. We thus could not differentiate between variation in adherence over time versus lack of reproducibility, and could not include a measure of adherence to restrictions. We assessed only symptomatic infections, and it is also likely, despite our efforts to ensure access to testing, including confidential study testing, that our case ascertainment was incomplete and may have been biased. Some participants, particularly later during the Omicron wave, may not have reported or been tested for mild symptoms. In addition, lower income participants unable to work from home may have been less willing to report symptoms or have even study testing performed. In our non-EI cohort, 49% (95% CI 38–60) of participants over the age of 60 years reported a confirmed infection by January 2023, significantly less than the 65% of Ontario adults exhibiting infection induced immunity at this date [15]. This suggests either that some infections in participants may have been missed or asymptomatic, or that our study population was more adherent to vaccination and public health measures and less likely to be infected than the general population. Although our two cohorts were matched on testing date and indication early in the pandemic and we attempted to adjust for other factors, we cannot rule out unmeasured effects. For instance, if individuals with EI were systematically less likely to report symptoms and/or be tested during Omicron we would over-estimate the protective effect of prior infection. However, it is notable that non-EI participants received more vaccine doses during the Omicron wave: if our multivariable adjustment for vaccine effect is incomplete, this would result in under-estimation of the effect of early infection. Our data are derived from a single jurisdiction with relatively low rates of infection pre-Omicron and high vaccination rates, and may not be applicable to other jurisdictions with differing risk of infection or different vaccine uptake. Our data apply to all infections; the impact of infection-derived and hybrid immunity on severe infections cannot be inferred from these data.

## Conclusions

In conclusion, COVID-19 infections early in the pandemic in our highly vaccinated population provided significant protection against re-infection more than 2 years later during the entire first year of the Omicron wave, with effect sizes similar across all subpopulations. Social determinants of health appeared less important as risk factors for Omicron as compared to early infections. Longer follow-up is needed to determine the duration of protection from early infection.

## Supplementary Information

Supplementary Material 1.

## Data Availability

The datasets analysed during the current study are available from the corresponding author on reasonable request.

## Abbreviations

CI: confidence interval
COVID-19: coronavirus disease of 2019
EI: early infection, defined as having laboratory confirmed COVID-19 before September 30, 2020
FT: full-time
HR: hazard ratio
NA: not available
NAAT: nucleic acid amplification test
NE: not evaluable
PCR: polymerase chain reaction
PT: part-time
RAT: rapid antigen test
SARS-CoV-2: Severe Acute Respiratory Syndrome Coronavirus 2
SE: southeast
RT-PCR: reverse transcriptase polymerase chain reaction

## References

[1] Reiner RC, Collins JK, Murray CJL. Forecasting the trajectory of the COVID-19 pandemic into 2023 under plausible variant and intervention scenarios: a global modelling study. medRxiv, 10.1101/2023030723286952. 2023.

[2] World Health Organization. Coronavirus (COVID-19) Dashboard. https://covid19.who.int/. Accessed August 6, 2023.

[3] Portmann L, De Kraker MEA, Fröhlich G, et al. Hospital outcomes of Community-Acquired SARS-CoV-2 Omicron variant infection compared with influenza infection in Switzerland. JAMA Netw Open. 2023;6(2):e2255599. 36790812 10.1001/jamanetworkopen.2022.55599 PMC9932839

[4] Goldstein E. Mortality associated with Omicron and influenza infections in France before and during the COVID-19 pandemic. Epidemiol Infect. 2023;1–22. 10.1017/S0950268823001358. 10.1017/S0950268823001358 PMC10540177 37622317

[5] World Health Organization. Interim statement on hybrid immunity and increasing population seroprevalence rates. June 1. 2022. https://www.who.int/news/item/01-06-2022-interim-statement-on-hybrid-immunity-and-increasing-population-seroprevalence-rates.

[6] Irving SA, Buchan SA. Considerations of hybrid immunity and the future of adolescent COVID-19 vaccination. Lancet Infect Dis. 2023;23:382–3. 10.1016/S1473-3099(22)00759-9. 36436535 10.1016/S1473-3099(22)00759-9 PMC9691141

[7] Stein C, Nassereldine H, Sorensen RJD et al. Past SARS-CoV-2 infection protection against re-infection: a systematic review and meta-analysis. Lancet. 2023; 401:833–842. https://linkinghub.elsevier.com/retrieve/pii/S0140673622024655. 10.1016/S0140-6736(22)02465-5 PMC9998097 36930674

[8] Bobrovitz N, Ware H, Ma X, et al. Protective effectiveness of previous SARS-CoV-2 infection and hybrid immunity against the omicron variant and severe disease: a systematic review and meta-regression. Lancet Infect Dis. 2023;23:556–67. 10.1016/S1473-3099(22)00801-5. 36681084 10.1016/S1473-3099(22)00801-5 PMC10014083

[9] Arabi M, Al-Najjar Y, Sharma O, et al. Role of previous infection with SARS-CoV-2 in protecting against omicron reinfections and severe complications of COVID-19 compared to pre-omicron variants: a systematic review. BMC Infect Dis. 2023;23:432. 10.1186/s12879-023-08328-3. 37365490 10.1186/s12879-023-08328-3 PMC10294418

[10] Altarawneh HN, Chemaitelly H, Hasan MR, et al. Protection against the Omicron variant from previous SARS-CoV-2 infection. N Engl J Med. 2022;387:1620–22. 35139269 10.1056/NEJMc2200133 PMC8849180

[11] Tan CY, Chiew CJ, Pang D et al. Effectiveness of bivalent mRNA vaccines against medically attended symptomatic SARS-CoV-2 infection and COVID-19-related hospital admission among SARS-CoV-2-naive and previously infected individuals: a retrospective cohort study. Lancet Infect Dis. 2023:S1473-3099(23)00373-0. 10.1016/S1473-3099(23)00373-0. 10.1016/S1473-3099(23)00373-0 37543042

[12] Virk A, Johnson MG, Roellinger DL, et al. Hybrid immunity provides protective advantage over vaccination or prior remote coronavirus Disease 2019 alone. Open Forum Infect Dis. 2023;10(5):ofad161. 10.1093/ofid/ofad161. 37180597 10.1093/ofid/ofad161 PMC10167982

[13] Public Health Ontario. Weekly Epidemiological Summary. SARS-CoV-2 Genomic Surveillance in Ontario, July 2, 2024. Toronto, Ontario, Canada. https://www.publichealthontario.ca/-/media/documents/ncov/epi/covid-19-sars-cov2-whole-genome-sequencing-epi-summary.pdf. Accessed July 8, 2024.

[14] Public Health Ontario. Respiratory virus tool: Covid-19 vaccines. https://www.publichealthontario.ca/en/Data-and-Analysis/Infectious-Disease/Respiratory-Virus-Tool. Accessed July 8, 2024.

[15] Covid-19 Immunity Task Force. Seroprevalence in Canada. https://www.covid19immunitytaskforce.ca/seroprevalence-in-canada. Accessed July 8, 2024.

[16] Hadley E, Yoo YJ, Patel S, Zhou A, Laraway B, Wong R, Preiss A, Chew R, Davis H, Brannock MD, Chute CG, Pfaff ER, Loomba J, Haendel M, Hill E. N3C and RECOVER consortia; Moffitt R. insights from an N3C RECOVER EHR-based cohort study characterizing SARS-CoV-2 reinfections and long COVID. Commun Med (Lond). 2024;4:129. 10.1038/s43856-024-00539-2. 38992084 10.1038/s43856-024-00539-2 PMC11239932

[17] Shigayeva A, Rudnick W, Green K, et al. Invasive pneumococcal disease among immunocompromised persons: implications for Vaccination Programs. Clin Infect Dis. 2016;62:139–47. 26354970 10.1093/cid/civ803

[18] Statistics Canada. Postal Code Conversion File Plus (PCCF+) Version 7 C, Reference Guide. 2019.

[19] Kuster SP, Coleman BL, Raboud J et al. Risk Factors for Influenza among Health Care Workers during 2009 Pandemic, Toronto, Ontario, Canada. Emerg Infect Dis. 2013; 19:606–615. http://wwwnc.cdc.gov/eid/article/19/4/11-1812_article.htm. 10.3201/eid1904.111812 PMC3647716 23631831

[20] Lavell AHA, Tijdink J, Buis DTP, Smulders YM, Bomers MK, Sikkens JJ. Why not to pick your nose: association between nose picking and SARS-CoV-2 incidence, a cohort study in hospital health care workers. PLoS ONE. 2023;18:e0288352. 10.1371/journal.pone.0288352. 37531335 10.1371/journal.pone.0288352 PMC10395815

[21] Dean AG, Sullivan KM, Soe MM, OpenEpi. Open Source Epidemiologic Statistics for Public Health, Version 3.01. 2013. Available at: www.OpenEpi.com. Accessed 13 July 2024.

[22] Ramjith J, Roes KCB, Zar HJ, Jonker MA. Flexible modelling of risk factors on the incidence of pneumonia in young children in South Africa using piece-wise exponential additive mixed modelling. BMC Med Res Methodol. 2021;21:17. 10.1186/s12874-020-01194-6. 33430789 10.1186/s12874-020-01194-6 PMC7802241

[23] Lenguerrand E, Whitehouse MR, Beswick AD, et al. Risk factors associated with revision for prosthetic joint infection after hip replacement: a prospective observational cohort study. Lancet Infect Dis. 2018;18:1004–14. 10.1016/S1473-3099(18)30345-1. 30056097 10.1016/S1473-3099(18)30345-1 PMC6105575

[24] Health Canada. COVID-19 signs, symptoms and severity of disease: A clinician guide. Available at: https://www.canada.ca/en/public-health/services/diseases/2019-novel-coronavirus-infection/guidance-documents/signs-symptoms-severity.html. Accessed 18 Dec 2024.

[25] Chin ET, Leidner D, Lamson L, et al. Protection against Omicron from Vaccination and previous infection in a prison system. N Engl J Med. 2022;387:1770–82. 10.1056/NEJMoa2207082. 36286260 10.1056/NEJMoa2207082 PMC9634863

[26] Carazo S, Skowronski DM, Brisson M, et al. Estimated Protection of prior SARS-CoV-2 infection against Reinfection with the Omicron variant among Messenger RNA-Vaccinated and nonvaccinated individuals in Quebec, Canada. JAMA Netw Open. 2022;5(10):e2236670. 10.1001/jamanetworkopen.2022.36670. 36239934 10.1001/jamanetworkopen.2022.36670 PMC9568797

[27] Yung CF, Pang D, Kam KQ, et al. BNT162b2 vaccine protection against omicron and effect of previous infection variant and vaccination sequence among children and adolescents in Singapore: a population-based cohort study. Lancet Child Adolesc Health. 2023;7:463–70. 10.1016/S2352-4642(23)00101-3. 37201540 10.1016/S2352-4642(23)00101-3 PMC10185330

[28] Babouee Flury B, Güsewell S, Egger T, et al. Risk and symptoms of COVID-19 in health professionals according to baseline immune status and booster vaccination during the Delta and Omicron waves in Switzerland-A multicentre cohort study. PLoS Med. 2022;19(11):e1004125. 36342956 10.1371/journal.pmed.1004125 PMC9678290

[29] Cauchi JP, Dziugyte A, Borg ML, et al. Hybrid immunity and protection against infection during the Omicron wave in Malta. Emerg Microbes Infect. 2023;12(1):e2156814. 36510837 10.1080/22221751.2022.2156814 PMC9817114

[30] Powell AA, Kirsebom F, Stowe J, et al. Protection against symptomatic infection with delta (B.1.617.2) and omicron (B.1.1.529) BA.1 and BA.2 SARS-CoV-2 variants after previous infection and vaccination in adolescents in England, August, 2021-March, 2022: a national, observational, test-negative, case-control study. Lancet Infect Dis. 2023;23:435–44. 10.1016/S1473-3099(22)00729-0. 36436536 10.1016/S1473-3099(22)00729-0 PMC10032664

[31] Link-Gelles R, Weber ZA, Reese SE, et al. Estimates of Bivalent mRNA vaccine durability in preventing COVID-19-Associated hospitalization and critical illness among adults with and without Immunocompromising conditions - VISION Network, September 2022-April 2023. MMWR. 2023;72:579–88. 37227984 10.15585/mmwr.mm7221a3 PMC10231940

[32] Tartof SY, Xie F, Yadav R, et al. Prior SARS-CoV-2 infection and COVID-19 vaccine effectiveness against outpatient illness during widespread circulation of SARS-CoV-2 Omicron variant, US Flu VE network. Influenza Other Respir Viruses. 2023;17:e13143. 10.1111/irv.13143. 37246146 10.1111/irv.13143 PMC10209645

[33] Wang L, Calzavara A, Baral S, et al. Differential patterns by Area-Level Social Determinants of Health in Coronavirus Disease 2019 (COVID-19)-Related mortality and Non-COVID-19 mortality: a Population-based study of 11.8 million people in Ontario, Canada. Clin Infect Dis. 2023;76:1110–20. 10.1093/cid/ciac850. 36303410 10.1093/cid/ciac850 PMC9620355

[34] Sundaram ME, Calzavara A, Mishra S et al. Individual and social determinants of SARS-CoV-2 testing and positivity in Ontario, Canada: a population-wide study. CMAJ 202;193:E723–34. 10.1503/cmaj.202608. 10.1503/cmaj.202608 PMC8177943 33906966

[35] Brakefield WS, Olusanya OA, White B, Shaban-Nejad A. Social determinants and indicators of COVID-19 among marginalized communities: a scientific review and call to Action for Pandemic Response and Recovery. Disaster Med Public Health Prep. 2022;17:e193. 10.1017/dmp.2022.104. 35492024 10.1017/dmp.2022.104 PMC9237492

[36] Government of Canada. Canada Emergency Response Benefit (CERB), status: closed. https://www.canada.ca/en/services/benefits/ei/cerb-application.html#h2.03. Accessed 13 July 2023.

[37] Government of Ontario. Archived—renting: changes during COVID-19 (coronavirus). https://www.ontario.ca/page/renting-changes-during-covid-19. Accessed 13 July 2023.

[38] Government of Ontario. Ontario extending COVID-19 paid sick days. 7 December 2021. https://news.ontario.ca/en/release/1001296/ontario-extending-covid-19-paid-sick-days. Accessed 13 July 2023.

[39] Ma H, Chan AK, Baral SD, et al. Which curve are we flattening? The disproportionate impact of COVID-19 among economically marginalized communities in Ontario, Canada, was unchanged from wild-type to Omicron. Open Forum Infect Dis. 2022;10(1):ofac690. 10.1093/ofid/ofac690. 36726534 10.1093/ofid/ofac690 PMC9879750

[40] Swail H, Murphy T, Buckeridge D. SARS-CoV-2 Seroprevalence in Canada. https://borealisdata.ca/dataset.xhtml?persistentId=doi:10.5683/SP3/LAEQ5L. Accessed 13 July 2024.

