# Supplementary material for: Early COVID-19 and protection from Omicron in a highly vaccinated population in Ontario, Canada: a matched prospective cohort study
Source: BMC Infect Dis. 2025 Feb 8;25:194. doi: 10.1186/s12879-024-10331-1 (PMC11806543; doi:10.1186/s12879-024-10331-1)
Supplement: Supplementary file 1 — Supplementary Material 1. [file 12879_2024_10331_MOESM1_ESM.docx]

**SUPPLEMENT: COVID-19 infection early in the pandemic and protection from Omicron infection in a highly vaccinated population in Ontario, Canada**

| Table of contents | Page |
| --- | --- |
| 1. SARS-CoV-2 activity and testing strategies in Toronto, Canada from March of 2020 to January of 2023  - Supplementary Figure 1: Number and incidence of COVID-19 cases and hospitalizations in Toronto, Canada, from March 2020 to January 2023 | 2 |
| 1. Supplementary Table 1: Characteristics of cohort participants included in and excluded from the analysis | 3 |
| 1. Supplementary Figures  - SFigure 2: Covid-19 Vaccination among study participants, by calendar time. - SFigure 3: Hazard ratios and confidence limits for factors associated with risk of COVID-19 during Omicron, Cox proportional hazards model. | 4 |
| 1. Study questionnaires  - Baseline questionnaire - Bi-weekly questionnaire - Illness questionnaire - Workplace questionnaire, November 2022 | 5-13  14-17  18  19 |
|  |  |
|  |  |

1. **SARS-CoV-2 activity and testing strategies in Toronto, Canada from March of 2020 to January of 2023**

In the greater Toronto area, Ontario, Canada, the first SARS-CoV-2 case was diagnosed on January 24, 2020. Cases and hospitalizations peaked in April, then declined to low levels by mid-June (see Supplementary Figure 1).

Testing was restricted by capacity limits of supplies until May 29, 2020, with only persons with symptoms compatible with COVID-19 who required hospitalization, were part of a long term care outbreak, or were outpatients who were health care workers, contacts of a laboratory-confirmed case, or travellers from outside Canada eligible for testing. PCR testing then became available with the goal of testing all potential cases. Rapid antigen testing (RAT) was very limited outside of long term care until February of 2021. Wide use of RAT started in the autumn of 2023. Polymerase chain reaction (PCR) testing capacity was overwhelmed restricted again as of December 31, 2021 to hospitalized cases, long term care residents, some outbreaks, and health care workers. From 9 February, 2022 to 30 January 2023, rapid antigen kits were provided for free to the population of Ontario at participating pharmacies and grocery stores.

**Supplementary Figure 1:** COVID cases and hospitalizations in Toronto, Canada, Mar2020-Jan 2023

Available at: <https://www.publichealthontario.ca/en/data-and-analysis/infectious-disease/covid-19-data-surveillance/covid-19-data-tool?tab=trends> (accessed June 7, 2023).

1.
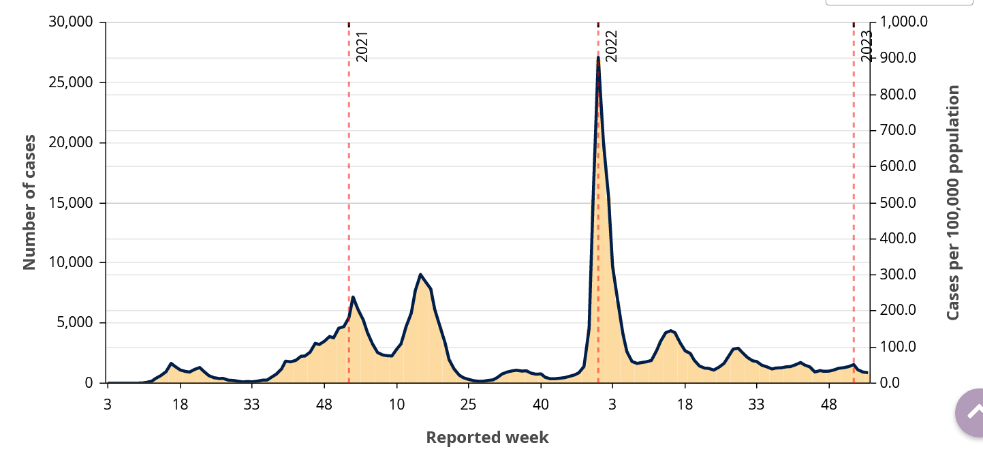
Cases
2.
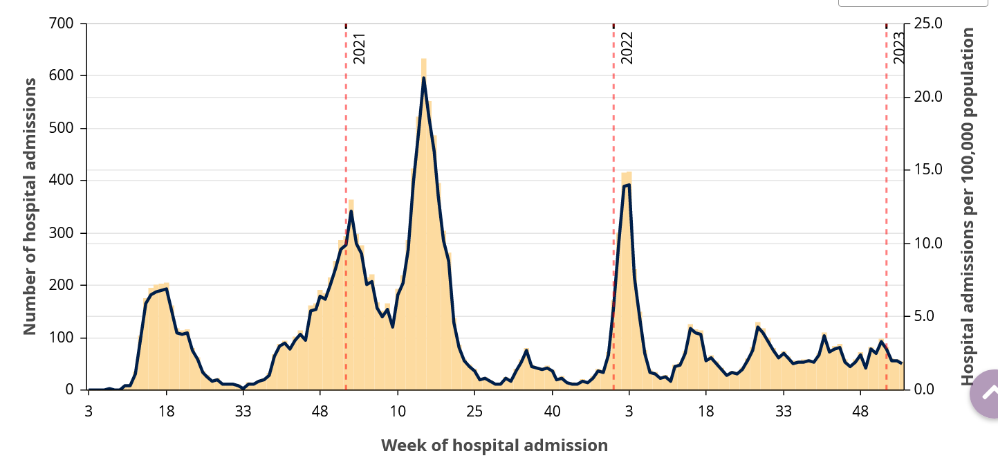
Hospitalizations
3. **Supplementary Table 1. Characteristics of enrolled participants included and excluded from the analysis**

|  | Total | Included  in analysis | Excluded | *P-value* |
| --- | --- | --- | --- | --- |
|  | N=1,124 | N=800 | N=324 |  |
| Study group |  |  |  |  |
| No early SARS-CoV-2 infection | 224 (19.9%) | 171 (21.4%) | 53 (16.4%) | 0.056 |
| Early SARS-CoV-2 infection | 900 (80.1%) | 629 (78.6%) | 271 (83.6%) |  |
| Sex at birth |  |  |  |  |
| Male | 543 (48.3%) | 376 (47.0%) | 167 (51.5%) | 0.167 |
| Female | 581 (51.7%) | 424 (53.0%) | 157 (48.5%) |  |
| Age (median, IQR) | 55 (39-65) | 56 (41.5 -66) | 51 (34-63) | <0.001 |
| Severity of 2020 illness |  |  |  |  |
| Inpatient | 533 (47.4%) | 370 (46.2%) | 163 (50.3%) | 0.217 |
| Outpatient | 591 (52.6%) | 430 (53.8%) | 161 (49.7%) |  |
| Neighbourhood income quintile |  |  |  |  |
| 1 - lowest | 361 (32.1%) | 241 (30.1%) | 120 (37.2%) | 0.05 |
| 2 | 262 (23.3%) | 183 (22.9%) | 79 (24.5%) |  |
| 3 | 191 (17.0%) | 140 (17.5%) | 51 (15.8%) |  |
| 4 | 133 (11.8%) | 97 (12.1%) | 36 (11.1%) |  |
| 5 - highest | 176 (15.7%) | 139 (17.4%) | 37 (11.5%) |  |
|  |  |  |  |  |
| Months in follow up (since 2020 test) | 32.8 (24.4-34.4) | 33.9 (32.4 - 34.7) | 20.8 (18.5 - 23.7) |  |
| SARS-CoV-2 episodes during follow up | 307 | 292 | 15 |  |
| Pre-Omicron | 18 | 15 | 3 |  |
| Omicron BA.1/BA.2 | 141 | 131 | 10 |  |
| Omicron BA.4.5 | 108 | 107 | 1 |  |
| Mixed BA.5/BQ1.1/XBB | 40 | 39 | 1 |  |

1. **Supplementary Fig 2. Covid-19 vaccination among study participants, by calendar week**


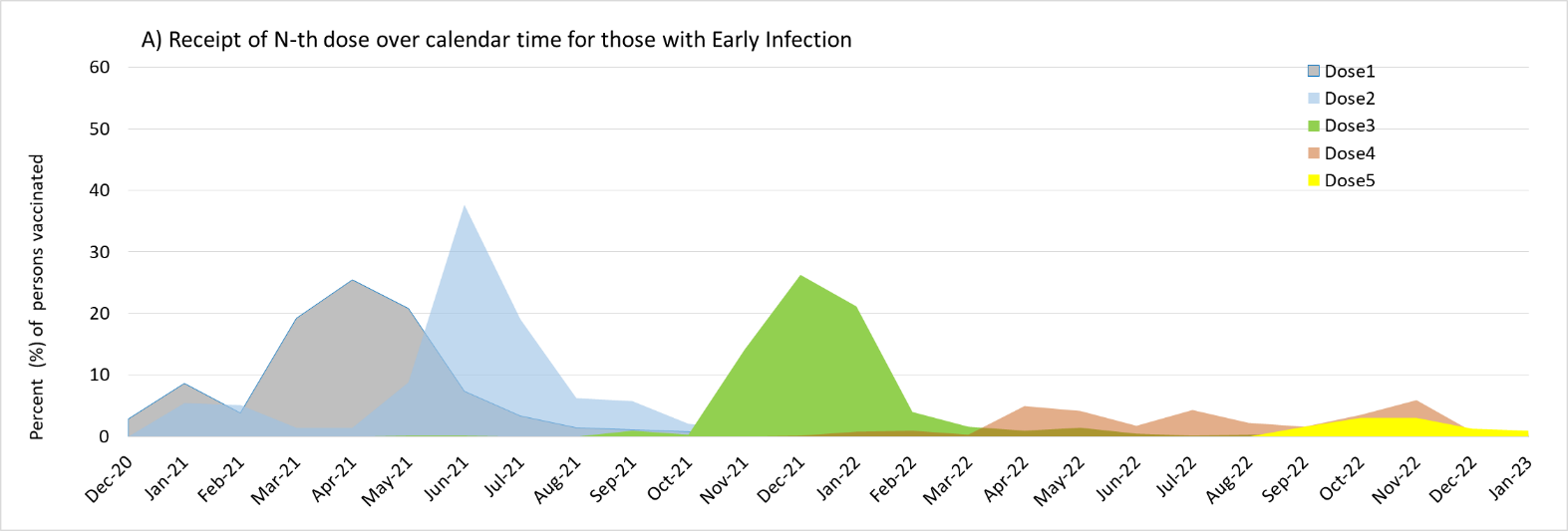


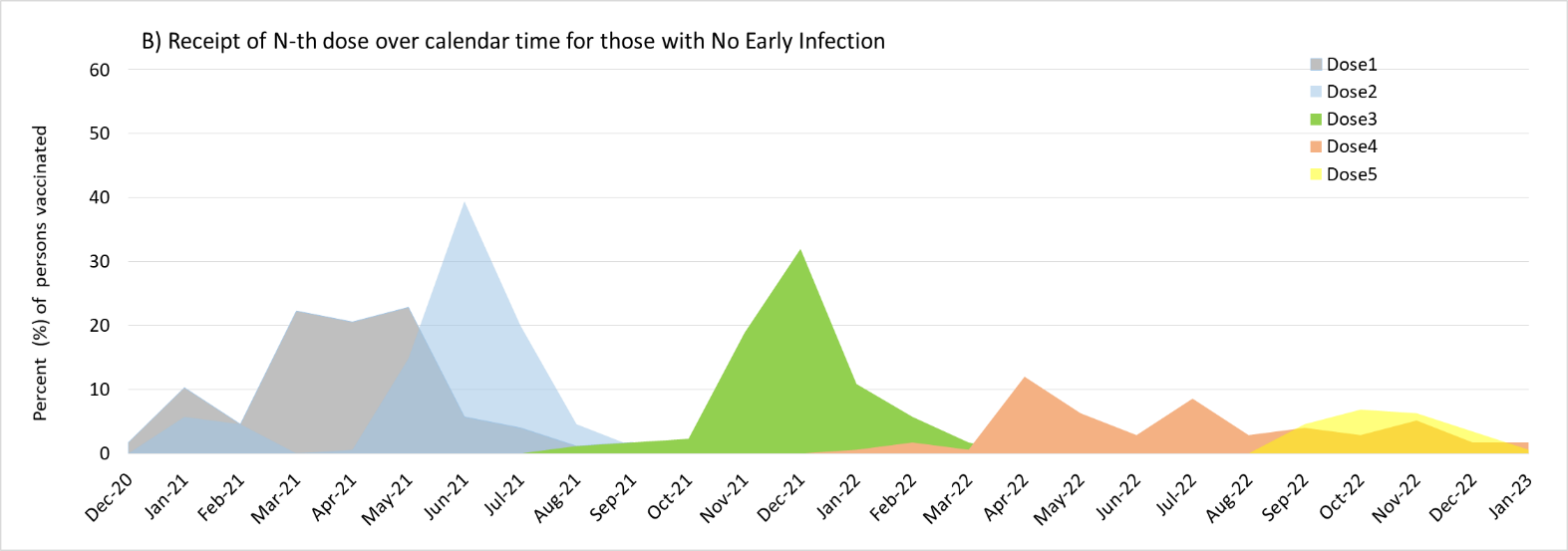


**Supplementary Figure 3:** Hazard ratios and confidence limits for factors associated with risk of COVID-19 during Omicron, Cox proportional hazards model.

# **Baseline Questionnaire: TIBDN RECOVER COVID-19 Cohort Study**

**Welcome!**

- **Thank you** for helping us learn more about COVID-19
- The following questions are to determine what might affect your risk for infection
- The questionnaire will take about 15 minutes to complete.
- You can start it now and if you get interrupted just scroll to the bottom, click the “SAVE” button, and come back later to finish it.
- If you have any questions about the survey, or about the study in general, please email: xxx or telephone xxx

**FIRST a few questions about your home and the people who live with you today**

1. What are the first 3 characters of the postal code of your home? __ __ __

2. How many people (other than yourself) live in your home? This includes people who sleep there at least 3 nights per week most weeks of the year. ______  *If 0, skip to Q#8*

3. How many bedrooms are there in your home or apartment? _________

4. How many other people sleep in the same room as you an average of 3 nights per week?

_______

5. How many children 17 years or younger live in your household (*who sleep there an average of 3 nights/week)*?

0 or ## ____

6. How many people 18 years or older (other than yourself) live in your household (*who sleep there an average of 3 nights/week)*  0 or ## ____

7. How many people in your household (other than yourself) usually worked, studied or volunteered for more than 7 hours per week outside the home before the **FIRST** COVID-19 restrictions (March 15, 2020)?

___ ##

7a. How many people in your household (other than yourself) worked, studied, or volunteered outside your home in the month after the restrictions/lockdown in March of 2020?

___ ##

7b. How many people in your household (other than yourself) currently work, study, or volunteer outside the home?

___ ##

7c. How many people in your household (other than yourself) work in an essential service? *(An essential service is one in which people have to go to work even if there is a lockdown – see attached for some examples)*

*_______*##

| *(Programmer: roll over of table for essential services)*Agriculture & food production |
| --- |
| Community services (sewage & water treatment, waste disposal) |
| Construction, maintenance, skilled trades |
| Consumer products (hardware, safety, vehicle sales, garden centres) |
| Financial services (banking, real estate, insurance) |
| Food (grocery, convenience, liquor, restaurant) |
| Health care, social services, education, research |
| Manufacturing, resources, energy, utilities |
| Services (pharmacy, gas station, delivery, funeral, vet, etc.) |
| Sports (professional) |
| Supply chain & transportation |
| Telecommunications & IT (including the media) |

8. Has anyone in your household (including you) spent at least one night outside of Canada since March 15, 2020 (the beginning of the March/school break last year)?

No

Yes 🡪 8a)

If yes: Where did you/they visit, what was the date of return to Ontario, and who travelled?

1. Country/province/territory visited: ______________

Date returned to Ontario: drop down calendar

Who travelled?  Just you

Yourself and others in your household

Just other household member(s)

(*programmer - if “i” completed, open “ii”, allow up to 5*

1. Country/province/territory visited: ______________

Date returned to Ontario: drop down calendar

Who travelled?  Just you

Yourself and others in your household

Just other household member(s)

9. Have you ever tested positive for COVID-19?

I have never been tested

No, tested negative

Waiting for result 🡪 What date were you tested? <DATE> (drop down calendar)

Yes 🡪 What date were you tested? <DATE> (drop down calendar)

*If you were tested more than once, please list the FIRST date that resulted in a positive test*

🡪 Have you tested positive again more than 3 months later? If yes, what date were you tested and

positive a second time?

<DATE> (drop down calendar)

*Programmer: please add reminder to complete illness questionnaire for each positive COVID-19 test*

10. Has anyone else in your household (not counting yourself) ever tested positive for COVID-19 (SARS-CoV-2)?

I live alone

No one has been tested

No, he/she tested negative

Not sure

Yes 🡪 When was the positive test? DATE> (drop down calendar for month and year only)

🡪 Were you tested after the exposure?

Yes, and I tested positive

Yes, and I tested negative

No, I was not tested

10a. Has anyone outside of your household, but with whom you have had close, unprotected contact within 2 meters and for 2 minutes or longer, tested positive for COVID-19?

No

Yes 🡪 Who tested positive? (*check all that apply)*

Family member

Friend

Co-worker

Patient that I cared for

Someone else: please list relationship: __________________________________

🡪 When was the positive test? Drop down calendar for month and year only

🡪 Were you tested after the exposure?

Yes, and I tested positive

Yes, and I tested negative

No, I was not tested

**A few questions about your health and vaccinations**

11. What is the month and year of your birth? ________________ _______ (drop down lists)

Month Year

12. Are you…

Female

Male

Non-binary, two-spirited or other

13. In general, would you say your health is…

Excellent

Very good

Good

Fair

Poor

14. Have you been diagnosed by a health professional with…

|  | Yes | No | Don’t Know |
| --- | --- | --- | --- |
| Asthma |  |  |  |
| COPD or other chronic lung condition |  |  |  |
| Diabetes |  |  |  |
| Heart disease |  |  |  |
| Cancer, that has been treated in the past 5 years |  |  |  |
| Liver or kidney disease |  |  |  |
| HIV/AIDS or other immune suppressing disease |  |  |  |
| Another long-term or chronic condition that has lasted – or is expected to last – at least 6 months: specify_______________________ |  |  |  |

15. Do you take any prescription medications (*not including birth control*)?

No

Yes 🡪 Please list the names of the medications: __________________________________________

*Programmer –provide space for 10*

16. Did you receive any of the following vaccines?

|  | Yes | No | Don’t Remember |
| --- | --- | --- | --- |
| Influenza, in the fall of 2020 |  |  |  |
| Influenza, in the fall of 2019 |  |  |  |
| Influenza, in the fall of 2018 |  |  |  |
| Influenza, in the fall of 2017 |  |  |  |
| Tetanus booster, in past 10 years |  |  |  |

17. Have you had at least one dose of a vaccine against COVID-19?

No – go to #18

Yes – please remember to complete your vaccination questionnaire! – skip to #19

18. Are you planning to be vaccinated against COVID-19?

Yes, I will take it - *go to #19*

Probably, but I need more information about how effective it is

Probably, but I need more information about side effects (safety)

Not likely, this year

Not likely, ever

Definitely not

*Any answer other than yes, skip to #20*

19. What were your reasons having been vaccinated or planning to be vaccinated? *(Check all that apply)*

I don’t want to get sick with COVID-19

I want to protect someone in my family from getting sick with COVID-19

I want to help stop the spread of COVID-19

I don’t want to wear a mask any longer

I want life to go back to normal (see friends, shop, etc.)

I want to travel

I want the economy to recover quickly

It is recommended by government officials (e.g., Health Canada, Public Health)

My physician recommended that I get it

Other, please specify: __________________________________

19a. If more than one answer to 19 What is the most important reason you were vaccinated or are planning to be vaccinated?

I don’t want to get sick with COVID-19

I want to protect someone in my family from getting sick with COVID-19

I want to help stop the spread of COVID-19

I don’t want to wear a mask any longer

I want life to go back to normal (see friends, shop, etc.)

I want to travel

I want the economy to recover quickly

It is recommended by government officials (e.g., Health Canada, Public Health)

My physician recommended that I get it

Other, please specify: __________________________________

20. If anything other than yes Q#18: What are your reasons for not planning to be vaccinated right away? *(Check all that apply)*

I don’t think I’ll get sick with COVID-19

I think I will have immunity because I was already infected

I don’t think I’ll get seriously ill if I am infected

I’m worried about possible side effects of the vaccine

I don’t think the vaccine will be very effective

I’m pregnant, breastfeeding, or hoping to get pregnant soon

I want to let people who are at higher risk than me get their vaccines first

I don’t belong to a group recommended for vaccination

Other, please specify: __________________________________

20a. If more than one answer to 20: What is the most important reason you don’t plan to be vaccinated right away?

I don’t think I’ll get sick with COVID-19

I think I will have immunity because I was already infected

I don’t think I’ll get seriously ill if I am infected

I’m worried about possible side effects of the vaccine

I don’t think the vaccine will be very effective

I’m pregnant, breastfeeding, or hoping to get pregnant soon

I want to let people who are at higher risk than me get their vaccines first

I don’t belong to a group recommended for vaccination

Other, please specify: __________________________________

**Now a few questions about your potential risk factors for COVID, and your preventive behaviours**

21. Do you wear prescription glasses?

No

All of the time

For both reading and distance, but not all of the time

For reading only

For distance only (e.g. driving)

Sometimes, but I usually wear contacts

22. How often do you use a travel mug/refillable drink container? This includes while at work, home, or during recreational activities.

Never

Less than once per month

Several times per month

Several times per week

Most days

23. How often do you rub your eyes or put your fingers or a pen/other objects in your mouth throughout the day?

Never

Once or twice per day

3-5 times per day

More than 5 times per day

24. How often do you bite your nails or cuticles, use your nails to clean your teeth, or pick your nose throughout the day?

Never

Once or twice per day

3-5 times per day

More than 5 times per day

25. In your lifetime, have you smoked a total of 100 or more cigarettes (about 4 packs), 50 cigars, or 50 pipes-full of tobacco in your life?

No

Yes 🡪 Do you currently smoke tobacco…

Daily

Occasionally

Not at all

26. Do you currently use e-cigarettes (vape) …

Daily

Occasionally

Not at all

27. Do you currently smoke or vape marijuana or cannabis (does not include edibles, oils, etc)…

Daily

Occasionally

Not at all

28. On days when you are not at work, how many times do you wash your hands (using soap and water or waterless hand rub)? *##*________ *(times/day)*

28a. On days when you are not at work, how many times do you come within 2 metres – for 2 minutes or longer - of someone outside of your household? *##________ (times/day)*

29. How often do you wear a mask when you are

|  | **Always** | **Usually** | **Occasion-ally** | **Rarely or**  **Never** | **I wear a face shield** | **Not applicable** |
| --- | --- | --- | --- | --- | --- | --- |
| In public place, like a store or on public transit |  |  |  |  |  |  |
| In a gym or fitness centre |  |  |  |  |  |  |
| In indoor spaces outside your home with friends or relatives from outside your immediate household |  |  |  |  |  |  |
| In indoor spaces in your home, with people from outside your immediate household |  |  |  |  |  |  |
| In your home, with only your own household members |  |  |  |  |  | (live alone) |
| At work, when you cannot maintain a distance of 2m from others |  |  |  |  |  | (work from home) |
| Outdoors, where physical distancing cannot be maintained |  |  |  |  |  |  |

30. In the past 14 days, did you attend any of the following activities in which there were five (5) or more people in attendance?

|  | Yes | No |
| --- | --- | --- |
| Meeting, conference (including at work) |  |  |
| Restaurant, bar, night club |  |  |
| Gathering of family or friends |  |  |
| Religious gathering, wedding, funeral, etc. |  |  |
| Gym, exercise class, team sport |  |  |
| Theatre, movie, opera, sporting event, casino |  |  |
| Other activity, please specify: ____________ |  |  |

31a. How do you feel overall about the public health measures that have been required in Ontario over the course of the pandemic

They have been far too stringent, and have caused much more harm than necessary

They have been somewhat too stringent, and have caused more harm than necessary

They have maintained about the best balance that could have been achieved between the threat of

COVID-19 and other harms

They have not been stringent enough, and more people have become ill and died than necessary

They have not been nearly stringent enough, and many more people have become ill and died than

necessary

31b. How have you adhered to the public health measures that have been required in Ontario (check the one answer that best reflects your life overall)

I am consistently more careful than the recommendations require

I am happy to have guidance, and generally follow the recommendations

I follow the recommendations even when I think they are more restrictive than necessary

I sometimes do things that are not recommended

I consistently do things that are not recommended

32. When you have done things that are not permitted by the public health/government guidance, what were the reason(s)? (check as many as applicable)

I have never done something not permitted/recommended

I was following the spirit of the guidance and not putting myself or others at risk

I was confused by the rules, and didn’t figure out until afterwards that I wasn’t supposed to do something

The activity was essential to me or to others

The restriction was unnecessary or unreasonable

Other, specify :

**The following sets of questions are about your work and workplace.**

33. What is your current work/study status?

I work full time – *go to #35*

I work part-time – *go to #35*

I am retired – *skip to #45*

I am a full or part time student at a school/college/university – *skip to #44*

I am a full time homemaker – *skip to #45*

I am not currently working or at school/college/university - *skip to #45*

34. What was your work/study status in February 2020 (before the pandemic)?

I was working full time

I was working part-time

I was retired

I was a full time homemaker

I was a full or part time student at a school/college/university

I was not currently working or at school/college/university

35. How many hours per week, on average, do you currently work? ____

Not applicable, I am not currently working, skip to #45

36. How many different jobs do you work at?

I have one full time job

I have one part-time job

I have one part-time and one full time job

I have two or more part time jobs

37. Please describe your usual work area currently. Do you usually (more than half of your time) work in an…

|  | Office by yourself |
| --- | --- |
|  | Office with co-workers &/or clients |
|  | Open area (e.g. hospital ward, grocery store) with co-workers &/or clients |
|  | No defined workspace (e.g. move throughout full hospital or store or building) |
|  | Not working, attending school, or volunteering outside the home (*skip to #44)* |
|  | Other: ______________________________ |

38. How has your usual work area changed since March 15, 2020?

Not at all, I have always worked from home *(skip to #45)*

I work from home now, doing work that was previously done in the workplace *(skip to #45)*

I still go into work and nothing has changed

I still go into work, but others are working from home

I still go into work, but we practice physical distancing and other protective measures

Other: please describe: _______________________________________________________

39. In your usual work area, how often do your colleagues practice good respiratory etiquette (keeping their distance from one another, cleaning hands frequently, & wearing a mask)?

Never

Occasionally

Usually

Always

Not applicable (work alone)

40. Please describe your colleagues’ approach to infectious respiratory illness (coughs, colds, influenza).

My colleagues stay home if they might be infectious to other people, even if they are

not that sick

My colleagues are at work unless they are too sick to be there

My colleagues come into work even if they are too sick to be productive

Not applicable (work alone)

41. Please describe your manager’s/supervisor’s approach to infectious respiratory illness in your workplace? [If you are the manager/supervisor, what is your approach?]

My manager encourages me to stay home if I might be infectious to other people, even if

I am not that sick

My manager expects that I will be at work unless I am too sick to be there

My manager expects me to be at work, no matter how bad I am feeling

Not applicable, I work alone

42. Do you have sick leave benefits? That is, are you paid for days that you are not at work because you are ill?

No

Yes

Don’t know

43. How many years have you been working in this occupation/profession?

<5 years

5-14 years

15+ years

44. Please indicate the description that best fits your attendance at school/college/university overall between March 2020 and June 2021

I attended only virtually – I was never in a room (classroom, lab etc.) with other students

I spent less than 25% of my class time in a room with other students

I spent 25-50% of my class time in a room with other students

I spent 50%-75% of my class time in a room with other students

I spent more than 75% of my class time in a room with other students

45. What is the highest level of education that you have ever attained?

Elementary school

Secondary school (grade 12 or 13/OAC)

Diploma or certificate from trade, technical or vocational school, college, or nursing school

Bachelor’s or undergraduate degree or teacher’s college (e.g., BA, BSc, LLB)

Master’s degree (e.g., MA, MSc, MEd)

Degree in Medicine, Dentistry, Optometry, Philosophy, etc.

Other – Specify: _______________________________

46. What is your cultural/racial background? Are you… *(check all that apply)*

White (European descent)

East Asian (Chinese, Japanese, Korean, Filipino, & others)

South Asian (East Indian, Pakistani, Sri Lankan, & others)

Southeast Asian (Cambodian, Indonesian, Loatian, Vietnamese, & others)

West Asian (Afghan, Iranian, Turkish, & others)

Middle East Asian/Arab (Egyptian, Iraqi, Lebanese, & others)

Black

Latin American

First Nations, Métis, Inuit, or other aboriginal

Other: specify: __________________________________________

Don’t know

46. Comments: _____________________________________________________________________________________

___________________________________________________________________________________________

**Thank you for helping us with this research study.**

**BI-WEEKLY REPORT – (5 of 6 reports)**

*Programmer note: If there has been an illness report filled out in the previous 14 days, then they do not need to fill in a bi-weekly report – they should NOT receive a reminder email to complete one and it should not be available on their dashboard

Report for the 2 week period ending on MONDAY: _____________ (automatically entered via website)

1. In the last 14 days, have you been tested for COVID-19?

No (go to #2)

Yes

Programmer note: These people will be taken directly to an illness report i.e., will not complete the remainder of the weekly report

1. In the last 14 days, have you had any of the following symptoms? *(check all that apply)*

|  | Fever |
| --- | --- |
|  | Cough (new/worsening) |
|  | Shortness of breath/difficulty breathing (new) |
|  | Generalized muscle aches/pains |
|  | Chills / shivering / feeling feverish |
|  | Feeling generally unwell or abnormally fatigued |
|  | Other: ____________________________________ |
|  | None of the above |

Programmer note: *If yes to cough, fever, or difficulty breathing (alone or with other symptoms) – AND - no illness report in past 14 days,* take them to an illness report (which they may/may not complete)

1. In the last 14 days, have you been exposed to a person with COVID-19?

No, or not that I am aware of

Yes, and I reported it to the study and have had my blood sample taken

Yes, please click HERE to report it

1. In the last 14 days, have you been vaccinated against COVID-19?

No

Yes, and I reported it to the study

Yes, please click HERE to report it

Programmer note: If they have completed COVID vaccination, please remove this question from the survey

<Submit button>

**“Extended” BI-WEEKLY REPORT – (1 of 6 reports)**

TIBDN RECOVER COVID study

*Programmer note: If there has been an illness report filled out in the previous 14 days, then they do not need to fill in a bi-weekly report – they should NOT receive a reminder email

Report for the two week period ending on MONDAY: _____________ (automatically entered via website)

1. In the last 14 days, have you been tested for COVID-19?

No (go to #2)

Yes 🡪 [These people will be taken directly to an illness report i.e., will not complete the remainder of the weekly report]

1. in the last 14 days, have you had any of the following symptoms? *Check all that apply*

|  | Cough (new/worsening) |
| --- | --- |
|  | Shortness of breath (new) |
|  | Chest pain (pressure/heaviness) |
|  | Feverishness, chills or shivering |
|  | Fever 🡪If yes, _ _ . _°C or _ _ _ ._^0^F or  not measured |
|  | Feeling generally unwell |
|  | Abnormally tired (new onset) |
|  | Confusion (new onset) |
|  | Generalized muscle aches/pains (new onset) |
|  | Joint pain (new onset) |
|  | Ear ache/infection |
|  | Headache (unusual or long lasting) |
|  | Runny or stuffy nose |
|  | Sinus pain |
|  | Sore/scratchy throat |
|  | Loss of appetite (new onset) |
|  | Nausea and/or vomiting |
|  | Diarrhea |
|  | Loss of taste and/or smell (new onset) |
|  | Other:__________________________________________ |
|  | Other: _________________________________________ |
|  | None of the above |

Programmer note: *If yes to cough, fever, or difficulty breathing (alone or with other symptoms) – AND - no illness report in past 14 days,* take them to an illness report (which they may/may not complete)

1. In the last 14 days, have you been exposed to a person with COVID-19?

No, or not that I am aware of

Yes, and I reported it to the study and have had my blood sample taken

Yes, please click HERE to report it

1. In the last 14 days, have you been vaccinated against COVID-19?

No

Yes, and I reported it to the study

Yes, please click HERE to report it

If yes,

4a. Did you have any side effects from the vaccine that were severe enough to interfere with your

activities of daily living, or required you to seek care from a health care provider?

No

Yes, and I reported it to the study

Yes, please click HERE to report it

Programmer note: If they have completed a COVID vaccination report, please remove this question from the survey

1. In the past 14 days, have you or anyone in your household returned from travel from outside of Ontario?

NO

YES

5a. If yes: Where did you/they visit, what was the date of return to Ontario, and who travelled?

1. Country/province/territory visited: ______________

Date returned to Ontario: drop down calendar

Who travelled?  Just you

Yourself and others in your household

Just other household member(s)

(if “i” completed, open “ii”)

1. Country/province/territory visited: ______________

Date returned to Ontario: drop down calendar

Who travelled?  Just you

Yourself and others in your household

Just other household member(s)

1. How many trips outside the home did you make in the past 14 days (work, school, shopping, healthcare, physical activity, visiting)?

___ drop down 0 to 30

6a. If >0, How many of those trips were on public transportation (bus, train, subway, etc.)?

___ drop down 0 to 42

7. Over the last 3 months, how often have you worn a mask when you were:

|  | **Always** | **Usually** | **Occasion-ally** | **Rarely or**  **Never** | **I wear a face shield** | **Not applicable** |
| --- | --- | --- | --- | --- | --- | --- |
| In public place, like a store or on public transit |  |  |  |  |  |  |
| In a gym or fitness centre |  |  |  |  |  |  |
| In indoor spaces outside your home with friends or relatives from outside your immediate household |  |  |  |  |  |  |
| In indoor spaces in your home, with people from outside your immediate household |  |  |  |  |  |  |
| In your home, with only your own household members |  |  |  |  |  | (live alone) |
| At work, when you cannot maintain a distance of 2m from others |  |  |  |  |  | (work from home) |
| Outdoors, where physical distancing cannot be maintained |  |  |  |  |  |  |

1. In the past 14 days, did you attend any the following activities in which there were 5 or more people who do not live in your household in attendance?

| Meetings, conferences (including work) |  |  |
| --- | --- | --- |
| Restaurant |  |  |
| Family gathering |  |  |
| School, classes, religious gatherings |  |  |
| Gym, exercise classes, team sports |  |  |
| Party, club/bar |  |  |
| Theatre, movie, opera, sporting event |  |  |
| Other activity |  |  |

1. How many times in the last 3 months have you been within 6 feet of a person outside of your household without wearing a mask?

Never or very rarely (<5 times)

Occasionally (less than once per week)

Sometimes (once per week to once per day)

Often (at least once per day

1. How do you feel overall about the public health measures that have been required in Ontario in the last 3 months

They have been far too stringent, and have caused much more harm than necessary

They have been somewhat too stringent, and have caused more harm than necessary

They have maintained about the best balance that could have been achieved between the threat of

COVID-19 and other harms

They have not been stringent enough, and more people have become ill and died than necessary

They have not been nearly stringent enough, and many more people have become ill and died than

necessary

11. How have you adhered to the public health measures that have been required in Ontario in the last three months (check the one answer that best reflects your life overall)

I am consistently more careful than the recommendations require

I am happy to have guidance, and generally follow the recommendations

I follow the recommendations even when I think they are more restrictive than necessary

I sometimes do things that are not recommended

I consistently do things that are not recommended

12. When you have done things that are not permitted by the public health/government guidance in the last 3 months, what were the reason(s)? (check as many as applicable)

I have never done something not permitted/recommended

I was following the spirit of the guidance and not putting myself or others at risk

I was confused by the rules, and didn’t figure out until afterwards that I wasn’t supposed to do something

The activity was essential to me or to others

The restriction was unnecessary or unreasonable

Other, specify :

Comments:

**ILLNESS REPORT**

TIBDN RECOVER COVID

What was the date of onset of your illness? Calendar drop down

Did you have a COVID test done during this illness? (please check as many as a many as applicable, if you had more than one test)

No,

Yes and I tested negative by PCR

Yes and I tested negative by Rapid antigen (RAT)

Yes and I tested positive by PCR

Yes and I tested positive by Rapid antigen (RAT)

I am not sure ( please comment in the box at the bottom of the page)

If you had a positive test (either RAT or PCR), what day was first positive swab collected? [*CLICK ON CALENDAR]*

*Note: If you were tested more than once, please list the FIRST date that resulted in a positive test associated with this illness*

1. How severe was this illness episode?

I tested positive but had no symptoms at all

I had symptoms, but was able to do regular activities on every day of the illness

I was not well enough to do regular activities on _______ days

I was not well enough to get out of bed for _______ days

I was admitted to the hospital for _______ days

Programmer note – if the participant reports no symptoms, subsequent questions should not appear

- If the participant reports being able to do regular activities on every day, Q5 should not appear

2. What care did you require?

None

One or more visits with a health care provider, virtual or in-person

One or more visits to an emergency department

I was admitted to the hospital

3. Did you have a fever?

No and I did not feel feverish

I felt feverish but did not take my temperature

Yes

If yes,  What was the highest temperature measured _________

On how many days did you have a temperature of more than 38ºC? ______ days

On what date were you first well enough to do regular activities? Calendar drop down or

Not yet completely recovered

On what date did you feel completely recovered? Calendar drop down or

Not yet completely recovered

Please write any additional comments about this illness episode:
